# The Sex-Determination Genes *fruitless* and *doublesex* Specify a Neural Substrate Required for Courtship Song

**DOI:** 10.1016/j.cub.2007.07.047

**Published:** 2007-09-04

**Authors:** Elizabeth J. Rideout, Jean-Christophe Billeter, Stephen F. Goodwin

**Affiliations:** 1 Division of Molecular Genetics, University of Glasgow, Anderson College, 56 Dumbarton Road, Glasgow G11 6NU, United Kingdom

**Keywords:** SYSNEURO

## Abstract

Courtship song is a critical component of male courtship behavior in *Drosophila*, making the female more receptive to copulation and communicating species-specific information [1–6]. Sex mosaic studies have shown that the sex of certain regions of the central nervous system (CNS) is critical to song production [7]. Our examination of one of these regions, the mesothoracic ganglion (Msg), revealed the coexpression of two sex-determination genes, *fruitless* (*fru*) and *doublesex* (*dsx*). Because both genes are involved in creating a sexually dimorphic CNS [8, 9] and are necessary for song production [10–13], we investigated the individual contributions of *fru* and *dsx* to the specification of a male CNS and song production. We show a novel requirement for *dsx* in specifying a sexually dimorphic population of *fru*-expressing neurons in the Msg. Moreover, by using females constitutively expressing the male-specific isoforms of *fru* (Fru^M^), we show a critical requirement for the male isoform of *dsx* (Dsx^M^), alongside Fru^M^, in the specification of courtship song. Therefore, although Fru^M^ expression is sufficient for the performance of many male-specific behaviors [14], we have shown that without Dsx^M^, the determination of a male-specific CNS and thus a full complement of male behaviors are not realized.

## Results and Discussion

Courtship behavior in *Drosophila melanogaster* consists of a sequence of behaviors performed by males to interest females in copulation. The male orients to the female, follows her, taps her abdomen with his foreleg, sings a species-specific courtship song, licks her genitals, attempts copulation, and finally copulates [15, 16]. Sex mosaic studies have shown that the sex of the central nervous system (CNS) is critical to the performance of these behaviors, suggesting that sex determination in the CNS is required for male sexual behavior in flies [7, 15, 17–21]. In particular, one sex-determination gene, *fruitless* (*fru*), is a key regulator of many steps in the courtship ritual [10, 12–14, 22].

Transcripts derived from the *fru* P1 promoter are spliced in females by the sex-specific splice factor Transformer (Tra) in conjunction with the non-sex-specific Transformer-2 (Tra-2), introducing a premature stop codon into female P1 transcripts. In males, a default splice occurs, giving rise to a class of male-specific *fru* isoforms (Fru^M^ proteins) [10, 13, 22–25] that are expressed in the CNS and peripheral nervous system (PNS) [23, 25–28] in regions associated with male-specific behaviors.

The constitutive expression of Fru^M^ isoforms in females triggers many male-specific courtship behaviors [14]. However, these females perform subnormal amounts of courtship and do not attempt copulation [14], suggesting that *fru* alone cannot specify all male courtship behaviors.

We examined the role of *doublesex* (*dsx*), another sex-determination gene, in the specification of male sexual behavior. *dsx* transcripts also undergo sex-specific splicing by Tra, producing male- and female-specific isoforms: Dsx^M^ and Dsx^F^, respectively [29, 30]. *dsx* is responsible for somatic sexual differentiation [15, 31–33] and aspects of sex-specific development in the CNS [8]. *dsx* is also expressed in the CNS and is necessary for wild-type courtship song in males [11, 34]. Recently, *dsx* was shown to act alongside *fru* in the differentiation of male-specific neurons in the abdominal ganglion [25]; however, few other studies have examined the relative contributions of both *fru* and *dsx* in specifying a male-specific CNS and regulating male sexual behavior. Therefore, this study examined the individual contributions of both genes in specifying courtship song.

### Fru^M^ Is Not Sufficient for Courtship Song

Courtship song in *Drosophila melanogaster* is male-specific and is critical to stimulating the female [4, 6]. It consists of a humming sound called sine song, and a rhythmically patterned pulse song, which together stimulate the female to mate, reducing the time to copulation [4, 6]. Pulse song also communicates species-specific information, allowing females to recognize conspecific males [1–3].

Fru^M^ mutant males lack pulse song [10, 12, 13], and constitutive Fru^M^ expression in the CNS of *fru*^M^ and *fru*^Δtra^ females induces the performance of many steps of the male courtship ritual [14], suggesting an important role for Fru^M^ in specifying courtship song. To determine the contribution of Fru^M^ in the specification of courtship song, we analyzed song production in females of genotype *fru*^M^ and *fru*^Δtra^.

**Figure 1:**
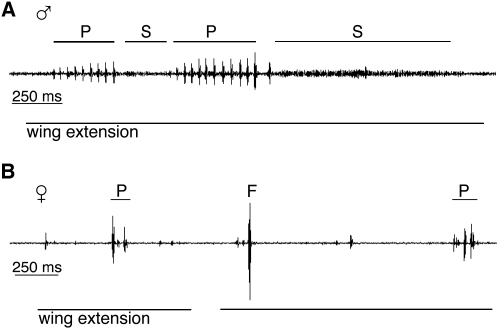
Representative Song Traces of *fru*^Δtra^ Males and Females Representative song traces from a 5–7-day-old *fru*^Δtra^*/Df(3R)fru^4-40^* male (A) and a *fru*^Δtra^*/Df(3R)fru^4-40^* female (B) produced in the presence of a single wild-type *Canton S* (CS) virgin female. Each song trace represents a fraction of a 5 min recording for a given individual. Wing extension during the recording is indicated below the song trace. Bouts of pulse song (“P”) and sine song (“S”) are recorded above the song trace. “F” indicates a noise caused by the CS target female falling.

Song analysis was based on 29 *fru*^M^ and *fru*^Δtra^ females because most *fru*^M^ and *fru*^Δtra^ females did not perform sufficient courtship behavior or song for analysis (total n = 61; Table S1 in the Supplemental Data available online). In accordance with [14], the wing-extension indices (WEIs) of these 29 *fru*^M^ and *fru*^Δtra^ females were not significantly different from wild-type and control *fru*^M^ and *fru*^Δtra^ males; however, we found a significant decrease in the song index (SI) (the percentage of time spent singing during wing extension) (p < 0.05) (Table S1). Also, the *fru*^M^ and *fru*^Δtra^ females' pulse song was highly aberrant (Figure 1). The number of pulse trains per minute (PTPM), mean pulses per train (MPPT) and interpulse interval (IPI) were all significantly lower compared to wild-type and control males (Table S1). Most striking, however, was the complete absence of sine song in these females (n = 29). Although the *fru*^M^ and *fru*^Δtra^ females were capable of wild-type wing extension, they spent significantly less time singing during courtship and produced song of poor quality. Thus, Fru^M^ expression alone cannot specify wild-type song production.

### Dsx^M^ Is Required to Specify Courtship Song

To dissect the individual contributions of both Fru^M^ and Dsx to the specification of courtship song, we analyzed males lacking Fru^M^ and Dsx (genotype *fru^3^,In(3R)dsx^23^/fru^3^,Df(3R)dsx^15^*). These double mutants had a courtship index (CI) of 0 toward females (n = 11) and no song. We have shown that Fru^M^ expression in females is not sufficient for courtship song. Likewise, the expression of Dsx^M^ in females is also not sufficient for song [35]. Thus neither *fru* nor *dsx* alone can specify courtship song. In fact, only the presence of both Fru^M^ and Dsx^M^, as in *transformer* (*tra*) mutant females, renders females capable of wild-type courtship song [36, 37], a finding we confirm. Together, these results demonstrate a previously unrecognized requirement for Dsx^M^, in conjunction with Fru^M^, in specifying courtship song.

### Dsx and Fru^M^ Colocalize in the CNS

Studies with male-female mosaics have shown that in gynandromorphs with a male head, the ventral thoracic ganglia of the adult CNS (including the mesothoracic ganglion [Msg]) must also be male for courtship song [7]. This suggests that the neural foci of courtship song are located in the ventral thoracic ganglia and that the sex of this region is critical to song. *fru* and *dsx* are both expressed in neurons located in this region, and mutations in both genes cause song defects [10–13, 23, 34]. In the abdominal ganglion (Abg) of the CNS, Fru^M^ and Dsx were shown to colocalize in a proportion of neurons and play critical roles in the development of male-specific clusters of serotonergic neurons [25]. Therefore, we asked whether Fru^M^ and Dsx were also coexpressed in the thoracic ganglia, and whether they act in parallel (if expressed in different neurons) or in concert (if expressed in the same neurons) to determine the neuronal substrate for courtship song in the CNS.

**Figure 2:**
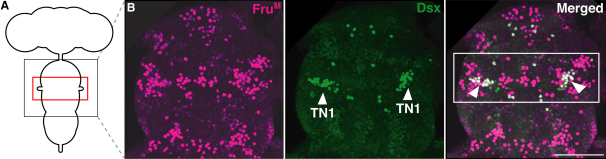
Colocalization of Fru^M^ and Dsx in the Mesothoracic Ganglion (A) Schematic representation of the CNS. The large black box indicates the region shown in the images, and the smaller red box surrounds the region of colocalization, the Msg. (B) Whole-mount ventral nerve cord (VNC) from 2-day-old pupal wild-type males colabeled with anti-Fru^M^ and anti-Dsx. The colocalization of Fru^M^ and Dsx in a subset of neurons in the TN1 cluster (arrowheads) of *dsx*-expressing neurons in the Msg of the VNC (white box) is shown. A ventral view is shown, with the anterior at the top. For the complete coexpression in the CNS, see Figure S1. The scale bar represents 50 μm.

We determined that Dsx and Fru^M^ colocalize in the Msg of the CNS (Figure 2B). Colocalization occurred in a subset of Dsx-expressing neurons (TN1 cluster; nomenclature as per [34]) from the pupal stage onward. The number of neurons coexpressing Dsx and Fru^M^ in 2-day-old male pupae was 17.4 ± 1.7 per hemisegment (mean ± standard deviation [SD]; n = 10), and was not significantly different in 5-day-old adults (p < 0.05). Colocalization occurred in a further two subsets of Dsx-expressing neurons in the posterior brain, pC1 and pC2 (nomenclature as per [34]), in addition to previously reported colocalization in the Abg [25] (Figure S1). Given the critical importance of the sex of the ventral ganglia (including the Msg) to song production, the colocalization of Fru^M^ and Dsx in this region suggests that sexually dimorphic developmental mechanisms might be operating in the Msg, contributing to the sex-specific nature of courtship song production.

### The Neuromuscular Junctions of the Direct Flight Muscles Are Not Sexually Dimorphic

Electrophysiological studies show that the activity of seven of the direct flight muscles (DFMs) is directly related to the beating of the wing during song [38]. These seven DFMs are the basalar muscles B1–B4, the anterior muscles of the first and third axillaries AX1a and AX3a, and the sternobasalar muscle SB (nomenclature as per [38]). The axonal morphology and cell-body location of the motor neurons innervating six of these DFMs (mnDFMs) has also been reported [39]. The cell bodies of these six mnDFMs lie in the ventral thoracic ganglia, five having cell bodies in the Msg [39]. We therefore investigated whether male-specific song production could be attributed to *fru*- and/or *dsx*-regulated sexually dimorphic characteristics of these motor neurons.

**Figure 3:**
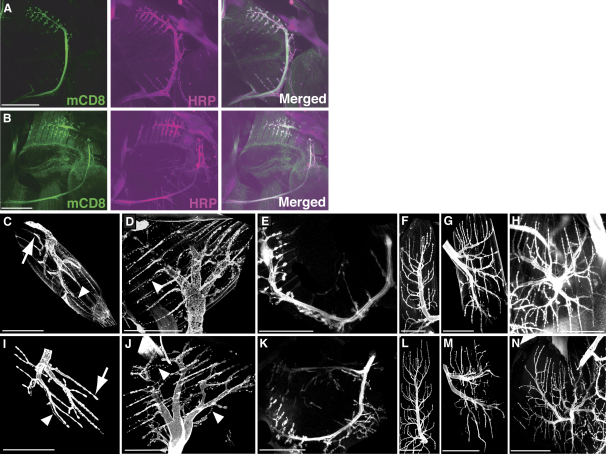
Motor Neurons Innervating the Direct Flight Muscles (A and B) *fru*^GAL4^ generates expression in a motor neuron innervating the direct flight muscles B3 and B4 (mnB3/B4). *fru*^GAL4^ was used to drive expression of the mouse lymphocyte (transmembrane) marker CD8 and green fluorescent fusion protein (*UAS-mCD8::GFP*) and was detected by anti-mCD8 labeling (mCD8; green). Anti-horseradish peroxidase (Anti-HRP) conjugated to Cy3 (HRP; purple) was used to reveal the neuronal projections of mnB3/B4 (purple) [48]. *fru*^GAL4^ drives expression in mnB3/B4 in both 5–7-day-old males (A) and females (B). (C–N) Axonal morphology of the motor neurons innervating the mnDFMs. The anti-HRP::Cy3 conjugate was used to reveal the neuronal projections [48]. Type I terminals (arrows) and type II terminals (arrowheads) are shown. Both types of synaptic terminals are present on all DFMs. The following axonal projections of mnDFMs from 5-day-old wild-type *Canton S* males were used: mnB1 (C), mnB2 (D), mnB3/B4 (E), mnAX1a (F), mnAX3a (G), and mnSB (H). The following axonal projections of mnDFMs from 5-day-old wild-type *Canton S* females were used: mnB1 (I), mnB2 (J), mnB3/B4 (K), mnAX1a (L), mnAX3a (M), and mnSB (N). The DFM nomenclature is as per [38]. Scale bars represent 50 μm.

First, we asked whether any of the mnDFMs were *fru* or *dsx* expressing. By using *fru*^GAL4^, a GAL4 driver expressing in all *fru* neurons [28], we determined that only mnB3/B4 (a single motor neuron innervating both B3 and B4 [39]) was *fru*^GAL4^ positive, and thus is a *fru* neuron (Figures 3A and 3B). This neuron was *fru*^GAL4^ positive in both males and females, and the innervation was not obviously sexually dimorphic. However, because some *dsx*-expressing neurons in the Msg are not *fru* expressing, we examined the axonal morphology of all mnDFMs to eliminate the possibility of sex-specific DFM innervation.

The axonal morphology and expression of common neurotransmitters at the neuromuscular junction (NMJ) of all seven DFMs were examined, and no obvious differences between the sexes were found (Figures 3C–3N). Type I and type II synaptic terminals were present on all mnDFMs, where type I terminals expressed glutamate and type II terminals expressed octopamine, in accordance with previous reports of neurotransmitter expression at the adult NMJ [40]. Moreover, no obvious differences in either axonal morphology or common neurotransmitters were observed in either *fru* or *dsx* mutant males (data not shown; genotypes are in the Supplemental Experimental Procedures). Therefore, the sexually dimorphic production of song is not likely to be a result of an obvious dimorphism in the neuronal morphology of the mnDFMs or in the neurotransmitter expression at the NMJs. Where might the critical difference(s) then lie?

### The Mesothoracic Ganglion Is Sexually Dimorphic

Kimura et al. [9] showed that Fru^M^ expression prevented *reaper*-mediated programmed cell death [41, 42] in a cluster of cells, resulting in more neurons in this cluster in males. Dsx^M^, on the other hand, prolongs neuroblast divisions in the Abg of males [8], again resulting in more neurons in males. Thus, sexual dimorphisms might be present in regions in which Dsx and Fru^M^ colocalize, as suggested by the ability of Fru^M^ and Dsx to generate sexually dimorphic neuronal populations [8, 9, 25]. Given that our investigation found no obvious sex-specific dimorphisms in the mnDFMs, the dimorphism might lie in a population of interneurons. Therefore, the Msg was examined so that it could be determined whether a sexually dimorphic population of neurons was present.

**Figure 4:**
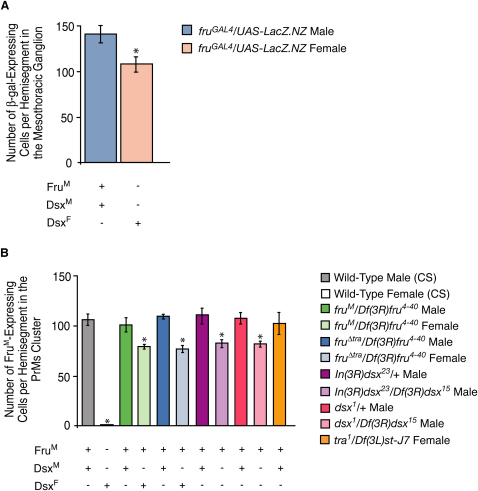
Sexually Dimorphic Neuron Numbers in the Mesothoracic Ganglion (A) Mean number of nuclei expressing β-Gal per hemisegment (±SD) in the Msg of the CNS in 5–7-day-old adult males and females. The mean number of nuclei is calculated from 10 hemisegments. *fru^GAL4^* drives the expression of β-Gal in both males and females. “^∗^” indicates a significant difference (p < 0.05). (B) Mean number of nuclei expressing Fru^M^ per hemisegment (±SD) in the PrMs cluster of Fru^M^-expressing neurons (nomenclature as per [23]) in 5–7-day-old adult flies. The mean number of nuclei per hemisegment is calculated from 10 hemisegments per genotype. “^∗^” indicates a significant decrease from wild-type and control males (p < 0.05). The presence or absence of Fru^M^, Dsx^M^, and Dsx^F^ is noted below each histogram bar.

By using *fru^GAL4^,* which expresses in both males and females [28], to drive a GAL4-responsive UAS-*LacZ*.NZ reporter, we quantified the number of β-Gal-positive neurons in males and females (Figure 4A). The number of β-Gal-positive neurons was significantly higher in males, with 136.4 ± 3.3 cells per hemisegment (n = 10) versus 111.6 ± 3.1 cells per hemisegment in females. Previously, Lee et al. [23] reported a sexual dimorphism in the number of neurons expressing *fru* P1 transcripts in the Msg. Together, these results suggest that a sexually dimorphic population of neurons is present in the Msg; therefore, we examined the individual contributions of Fru^M^ and Dsx in the creation of this difference in *fru*^GAL4^-positive neuron number in the Msg.

### Fru^M^ Expression Alone Cannot Create a Sexually Dimorphic Msg

We found a sexually dimorphic number of *fru*^GAL4^-expressing neurons in the Msg, a region of the CNS central to song production [7] and in which Fru^M^ and Dsx colocalize. To determine the individual contributions of *dsx* and *fru* in the creation of this sexually dimorphic number of neurons, we examined *fru*^M^ and *fru*^Δtra^ females to see if Fru^M^ expression alone abolishes the observed difference in neuronal number in the Msg between the sexes. We found that the number of Fru^M^-expressing neurons in the Msg of these females was significantly reduced in comparison to wild-type and control males (Figure 4B; Table S2). Furthermore, this decrease in Fru^M^-expressing neurons was comparable to the difference in neuron number observed in the Msg of *fru*^GAL4^ males and females driving the UAS-*LacZ*.NZ reporter.

Our results demonstrate that the difference in neuronal populations of males and females in the Msg lies in a subpopulation of Fru^M^-expressing neurons, and that Fru^M^ expression alone cannot eliminate this difference. Thus Fru^M^ expression cannot, by itself, dictate the creation of the sexually dimorphic population of neurons in the Msg. We therefore asked whether Dsx, which colocalizes with Fru^M^ in the Msg, plays a role in the specification of this sexually dimorphic population of neurons, helping to determine the full complement of Fru^M^ neurons.

### Dsx^M^ Is Required for Wild-Type Fru^M^ Expression

*dsx* affects the sex-specific development of other regions of the CNS [8, 25]. To determine whether *dsx* contributes to creating the sex-specific population of neurons in the Msg, we tabulated the number of Fru^M^-expressing neurons in the Msg of *dsx* null and *dsx* heterozygote control males. We found that *dsx* mutant males had significantly fewer Fru^M^-expressing neurons in the Msg than did wild-type and control males, demonstrating that Dsx is indeed required to obtain a full complement of Fru^M^-expressing neurons (Figure 4B; Table S2). Because *fru*^M^ and *fru*^Δtra^ females (who express the female-specific isoform of *dsx*, Dsx^F^) do not have a full complement of Fru^M^-expressing neurons in the Msg, we have demonstrated a critical role for Dsx^M^ in the creation of a sexually dimorphic Msg. In fact, only when both Fru^M^ and Dsx^M^ are present, as in *tra* mutant females, can a full complement of Fru^M^-expressing neurons in the Msg be obtained (Figure 4B). Thus, we have demonstrated a previously unrecognized requirement for Dsx^M^ in the specification of a population of Fru^M^-expressing neurons in the Msg.

Dsx^M^ prolongs the division of neuroblasts in the Abg of males, resulting in more neurons in the male Abg [8]. Also in the Abg, Dsx^M^ plays a critical role alongside Fru^M^ in the differentiation of a male-specific serotonergic population of neurons [25]. Our findings suggest that Dsx^M^ operates in a similar manner in the Msg and the posterior brain (E.J.R. and S.F.G., unpublished data) to create sexually dimorphic neuronal numbers. These differences in neuronal populations suggest a common developmental theme in colocalization regions, where Dsx^M^ generates a sexually dimorphic population of neurons, which is exploited by Fru^M^ to fashion a male-specific behavioral neural network [25] (cf. [9, 15]).

It is not clear why the absence of a sexually dimorphic population of Fru^M^-expressing neurons in the Msg is associated with striking defects in courtship song because our results suggest that this population of Fru^M^-expressing neurons does not directly innervate the DFMs. We propose that the Fru^M^-expressing neurons form at least part of a male-specific neural network responsible for controlling the production of courtship song.

Thus, although Fru^M^ expression can specify many male-specific behaviors, we show that without Dsx^M^, the determination of a complete male-specific CNS, and therefore a full complement of male behaviors, is not realized. This additional gene function is critical to understanding complex sex-specific phenotypes compared to previous interpretations of function, where *fru* has been described as the only gene needed for a “genetic switch” to male sexual behavior in *Drosophila*. Significantly, it adds to the growing evidence that *fru* and *dsx* are both necessary for a complete male courtship repertoire, in both neural and nonneural tissues [11, 25, 34, 43–47].

## Supplemental Data

Document S1. Experimental Procedures, One Figure, and Two Tables
