# Supplementary material for: The Sex-Determination Genes fruitless and doublesex Specify a Neural Substrate Required for Courtship Song
Source: Curr Biol. 2007 Sep 4;17(17-3):1473–8. doi: 10.1016/j.cub.2007.07.047 (PMC2583281; doi:10.1016/j.cub.2007.07.047)
Supplement: Document S1. Experimental Procedures, One Figure, and Two Tables [file mmc1.pdf]

Supplemental Data

The Sex-Determination Genes *fruitless* and *doublesex* Specify a Neural Substrate Required for Courtship Song

Elizabeth J. Rideout, Jean-Christophe Billeter, and Stephen F. Goodwin

Supplemental Experimental Procedures

D. melanogaster Strains and Crosses

Stocks and genetic crosses are as per [S1, S2]. Wild-type flies were obtained from a *Canton S* (CS) stock. fru^{M} and $fru^{\Delta tra}$ alleles were examined in *trans* to $Df(3R)fru^{4\text{-}40}$ as per [S3]. The fru^{GAL4} line [S4] was crossed to the nuclear-localized β-Galactosidase *UAS-LacZ*.NZ (line J312) reporter line or the membrane-targeted green fluorescent protein (GFP) reporter *UAS-mCD8::GFP* [S5], obtained from the *Drosophila* Stock Center (Bloomington, Indiana). *dsx* null mutants were heterozygous combinations of $Df(3R)dsx^{15}$ with either $In(3R)dsx^{23}$ or dsx^{1} (as per [S6]). *tra* nulls are heterozygous combinations of tra^{1} and *Df(3L)st-J7* (as per [S7]). Haplo-X progeny were distinguished from diplo-X with a *Bar*-marked Y chromosome. Fru^{M} mutants were fru^{3} homozygotes. The *fru,dsx* double mutants were $In(3R)dsx^{23},fru^{3}/Df(3R)dsx^{15},fru^{3}$.

Immunocytochemistry

All samples involving Fru^{M} and anti-β-Gal labeling were dissected and processed as per [S8] and [S1], respectively. Fru^{M} rabbit antibody was used as described in [S7]. Anti-Dsx labeling experiments were as per [S9]. anti-β-Gal (1:1000), anti-Fru^{M} (1:300), anti-Dsx (1:300), and anti-rabbit- and anti-rat-conjugated Alexa Fluor secondaries (1:600) (Invitrogen, Molecular Probes) were used. Anti-mCD8alpha (mCD8; Caltag Laboratories) was used at dilution of 1:20 and detected with anti-rat Alexa Fluor 488 (as per [S1, S7]). Images were acquired with a Zeiss LSM 510 Meta confocal microscope. All cell counts were performed on slides coded by an unbiased third party, and the cell numbers tabulated as per [S1].

mnDFM Preparations

Flies for dissection were collected after eclosion and aged in groups for 5–7 days. Flies were dissected in phosphate-buffered saline (PBS) and fixed in 4% (w/v) paraformaldehyde (in PBS) for 30 min at room temperature. The direct flight muscles (DFMs) were isolated (see drawing in [S10]), and Cy3-conjugated anti-horseradish peroxidase (HRP; Jackson ImmunoResearch Laboratories) was applied to the preparations at a dilution of 1:300. Preparations were mounted and visualized as per [S1].

Behavior

Male flies and fru^{M} and $fru^{\Delta tra}$ females were collected soon after eclosion and aged individually for 7 days prior to testing. Males were introduced into a round courtship chamber (1 cm in diameter × 4 mm height) with a 1-day-old wild-type virgin female and placed inside an Insectavox for recording [S11]. Recordings lasted 5–10 min. Audio and video recordings were captured with a Sony DCR-VX2100E digital camera (as per [S2]). Recordings were converted and compressed with FootTrack 2.3.2 software (T-squared Software [www.foottrack.com]), and behaviors were logged and analyzed with LifeSongX software (http://lifesong.bio.brandeis.edu) so that behavioral parameters could be calculated (as per [S2]). Courtship index (CI) and wing-extension index (WEI) were logged as per [S2]. Singing index (SI) was defined as the percentage of time spent singing during wing extension. Song parameters measured were the number of sine song bouts per minute (SBPM) calculated as (total bouts/total secs) × 60 s; the number of pulse trains per minute (PTPM), calculated as for SBPM; mean pulses per train (MPPT), where a minimum threshold of 2 pulses per train was set; and the interpulse interval (IPI).

Statistics

Behavioral parameters (CI, WEI, and SI) were subjected to arcsine-square-root transformations so that normality could be approximated, as per [S6]. Transformed values for behavioral parameters, along with untransformed values for pulse-song parameters and cells counts were subjected to one-way analysis of variance (ANOVA). Statistical tests were performed with JMP v6.0 software (SAS Institute).

Supplemental References

S1. Billeter, J.C., and Goodwin, S.F. (2004). Characterization of *Drosophila fruitless*-Gal4 transgenes reveals expression in male-specific *fruitless* neurons and innervation of male reproductive structures. J. Comp. Neurol. *475*, 270–287.

S2. Villella, A., Ferri, S.L., Krystal, J.D., and Hall, J.C. (2005). Functional analysis of *fruitless* gene expression by transgenic manipulations of *Drosophila* courtship. Proc. Natl. Acad. Sci. USA *102*, 16550–16557.

S3. Demir, E., and Dickson, B.J. (2005). *fruitless* splicing specifies male courtship behavior in *Drosophila*. Cell *121*, 785–794.

S4. Stockinger, P., Kvitsiani, D., Rotkopf, S., Tirian, L., and Dickson, B.J. (2005). Neural circuitry that governs *Drosophila* male courtship behavior. Cell *121*, 795–807.

S5. Lee, T., and Luo, L. (1999). Mosaic analysis with a repressible cell marker for studies of gene function in neuronal morphogenesis. Neuron *22*, 451–461.

S6. Villella, A., and Hall, J.C. (1996). Courtship anomalies caused by *doublesex* mutations in *Drosophila melanogaster*. Genetics *143*, 331–344.

S7. Billeter, J.C., Villella, A., Allendorfer, J.B., Dornan, A.J., Richardson, M., Gailey, D.A., and Goodwin, S.F. (2006). Isoform-specific control of male neuronal differentiation and behavior in *Drosophila* by the *fruitless* gene. Curr. Biol. *16*, 1063–1076.

S8. Lee, G., Foss, M., Goodwin, S.F., Carlo, T., Taylor, B.J., and Hall, J.C. (2000). Spatial, temporal, and sexually dimorphic expression patterns of the *fruitless* gene in the *Drosophila* central nervous system. J. Neurobiol. *43*, 404–426.

S9. Lee, G., Hall, J.C., and Park, J.H. (2002). *doublesex* gene expression in the central nervous system of *Drosophila melanogaster*. J. Neurogenet. *16*, 229–248.

S10. Trimarchi, J.R., and Schneiderman, A.M. (1994). The motor neurons innervating the direct flight muscles of *Drosophila melanogaster* are morphologically specialized. J. Comp. Neurol. *340*, 427–443.

S11. Gorczyca, M., and Hall, J.C. (1987). The Insectavox, an integrated device for recording and amplifying courtship songs of *Drosophila*. Drosophila Information Service *66*, 157–160.

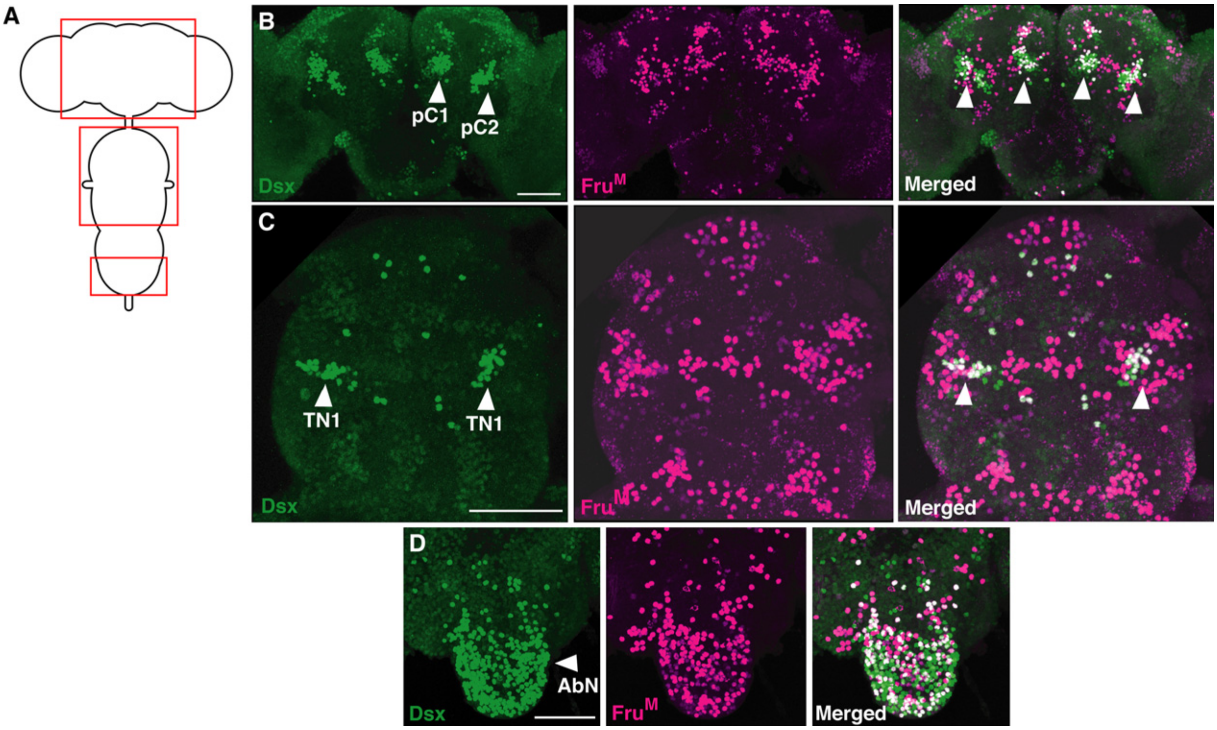

Figure S1. Colocalization of Fru^M and Dsx in the Central Nervous System

(A) Schematic representation of the CNS. Red boxes (top to bottom) outline the corresponding regions in the CNS from which the accompanying confocal images were taken.

(B–D) Whole-mount confocal images from 2-day-old pupal wild-type males colabeled with anti-Fru^M and anti-Dsx. Colocalization was seen in four distinct regions of the CNS: a subset of neurons in the pC1 and pC2 clusters of *dsx*-expressing neurons in the posterior brain, where 34 ± 4.6 neurons per hemisegment in pC1 and 22.8 ± 5.6 neurons per hemisegment in pC2 showed colocalization (B), in a subset of neurons in the TN1 cluster of *dsx*-expressing neurons in the Msg (C), and in a subset of the AbN cluster of *dsx*-expressing neurons in the abdominal ganglion (D) (nomenclature as per [S9]). (B) shows a dorsal view with anterior at the top. (C) and (D) show a ventral view with anterior at the top. The scale bar represents 50 μm.

Table S1. Song Analysis of Wild-Type and Mutant Flies.

Genotype	n	WEI	SI	SBPM	PTPM	MPPT	IPI (ms)
Canton S (XY)	15	38.3 ± 2.8	87.9 ± 2.4	18.6 ± 2.2	19.9 ± 1.4	8.1 ± 0.3	31.7 ± 3
XY;;fru^M/$Df(3R)fru^{4-40}$	15	53.0 ± 2.9	90.3 ± 2.0	23.1 ± 1.9	28.5 ± 2.3	9.1 ± 0.3	32.0 ± 3
XY;;$fru^{\Delta tra}$/$Df(3R)fru^{4-40}$	18	54.6 ± 2.8	85.3 ± 2.2	20.6 ± 1.8	29.3 ± 1.8	9.6 ± 0.4	31.7 ± 4
XX;;fru^M/$Df(3R)fru^{4-40}$	16	37.6 ± 3.3	44.8 ± 4.7*	0*	7.1 ± 1.5*	3.0 ± 0.1*	26.5 ± 7*
XX;;$fru^{\Delta tra}$/$Df(3R)fru^{4-40}$	13	31.4 ± 2.9	60.1 ± 5.6*	0*	9.4 ± 1.4*	2.9 ± 0.1*	24.3 ± 5*
XX;;tra^1/*Df(3L)st-J7*	10	46.3 ± 4.5	89.7 ± 2.9	23.2 ± 1.8	19.2 ± 1.4	9.0 ± 0.2	33.0 ± 3
XY;;$In(3R)dsx^{23}$,fru^3/ $Df(3R)dsx^{15}$,fru^3	11	0*	0*	N.D.	N.D.	N.D.	N.D.

Analysis of behavioral and song parameters of 5–7-day-old adults expressing different combinations of Fru^M, Dsx^M, and Dsx^F. "n" indicates the number of flies included in the analysis per genotype. Data for wing extension index (WEI), song index (SI), sine bouts per minute (SBPM), and pulse trains per minute (PTPM) are shown as mean ± standard error of the mean (SEM). Mean pulses per train (MPPT) and interpulse interval (IPI) values represent the mean of n intramale means for each genotype. "*" indicates a significant decrease from wild-type and control males ($p < 0.05$). "N.D." indicates no data because of the absence of song to analyze. A total of 61 fru^M and $fru^{\Delta tra}$ females were recorded, of which seven had a courtship index (CI) of < 10% (class 1), 25 with a CI > 10% but an SI < 10% (class 2), and finally, 29 with an SI > 10% (class 3). Only the class 3 fru^M and $fru^{\Delta tra}$ females were included in the analysis of courtship behavior and song.

Table S2. Quantifying Fru^M-Expressing Neurons in the Mesothoracic Ganglion.

Genotype	Fru^M cells per hemisegment (±SD)
Canton S (XY)	105.4 ± 5.5
Canton S (XX)	0 ± 0*
XY;;*fru^M/Df(3R)fru^4-40* → $XY;;fru^M/Df(3R)fru^{4\text{-}40}$	102.3 ± 7.2
$XX;;\ fru^M/Df(3R)fru^{4\text{-}40}$	75.3 ± 3.7*
$XY;;\ fru^{\Delta tra}/Df(3R)fru^{4\text{-}40}$	110.3 ± 4.8
$XX;;\ fru^{\Delta tra}/Df(3R)fru^{4\text{-}40}$	73.1 ± 4.6*
$B^sY;;In(3R)dsx^{23}/+$	106.4 ± 5.0
$B^sY;;In(3R)dsx^{23}/Df(3R)dsx^{15}$	82.6 ± 4.4*
$B^sY;;dsx^1/+$	107.3 ± 6.5
$B^sY;;dsx^1/Df(3R)dsx^{15}$	82.1 ± 2.8*
$XX;;tra^1/Df(3L)st\text{-}J7$	101.7 ± 5.6

Mean number of nuclei expressing Fru^M per hemisegment (±SD) in the PrMs cluster of Fru^M-expressing neurons (nomenclature as per [S8]) in 5–7-day-old adults. Ten hemisegments per genotype were used to calculate the mean number of nuclei. *dsx* and *tra* mutant males were distinguished by having *Bar-Stone* eyes (B^S-marked Y chromosome). "*" indicates a significant decrease from wild-type males ($p < 0.05$).
